# A Treatise on the Diseases of the Nervous System

**Published:** 1886-12

**Authors:** 


### Book Reviews.

A TREATISE ON THE DISEASES OF THE NERVOUS SYSTEM. By William A. Hammond, M. D., Surgeon-General U. S. Army (retired list); Professor of Diseases of the Mind and Nervous System in New York; Post-graduate Medical School and Hospital; Member of the American Neurological Association and of the New York Neurological Society; of the New York County Medical Society; of the New York Medico-Legal Society; of the American Philosophical Society (Philadelphia); of the Academy of the Natural Sciences (Philadelphia); Fellow of the College of Physicians (Philadelphia); Fellow of the American Academy of Arts and Sciences (Boston); Corresponding Member of the Anthropological Institute of Great Britain and Ireland; Honorary Member of the Royal Medico-Chirurgical Society of Edinburg; of the British Medical Association, etc., etc. With one hundred and twelve illustrations. Eighth edition, with corrections and additions. New York: D. Appleton & Co., 1, 3 and 5 Bond street. 1886.

An introductory chapter of this work describes the instruments and apparatus employed in the diagnosis and treatment of diseases of the nervous system, and explains the methods by which they are used. These means of investigating the various nervous derangements to which the human organization is subject have done much toward their methodic treatment, and have paved the way for further knowledge in this field.

The book is divided into seven sections, comprising Diseases of the Brain, filling 358 pages; Diseases of the Spinal Cord, with 273 pages; Cerebro-Spinal Diseases, with 157 pages; diseases of the peripheral nervous system, with 100 pages; and the addition of a new section on certain obscure diseases of the nervous system, which brings the work up to date.

It is a thorough disquisition upon the essentials of that complexity in the details of nervous disorders included under the designation of Diseases of the Nervous System, and the matured experience of the author after observing and treating a great variety of cases renders his revision of much value in that class of affections considered in this treatise.

Not only have great advances been made in our knowledge of the abnormalities of the functions of the nerve centres, and the modification in the action of the various ramifications of the nerves in different regions, but progress has been made in the etiology of disease based upon the impairment of nerve power, so that even practical surgery has profited greatly by neurology within the past few years.

The high estimation in which the former editions of the work have been held by the profession guarantees an intelligent appreciation of this new edition of superior merit.

